# Interleukin-6 receptor blockade or TNFα inhibition for reducing glycaemia in patients with RA and diabetes: post hoc analyses of three randomised, controlled trials

**DOI:** 10.1186/s13075-020-02229-5

**Published:** 2020-09-09

**Authors:** Mark C. Genovese, Gerd R. Burmester, Owen Hagino, Karthinathan Thangavelu, Melitza Iglesias-Rodriguez, Gregory St John, Miguel A. González-Gay, Thomas Mandrup-Poulsen, Roy Fleischmann

**Affiliations:** 1 Division of Immunology and Rheumatology, Stanford University Medical Center, 1000 Welch Road, Suite 203, Palo Alto, CA 94304 USA; 2 Charité University Medicine, Free University and Humboldt University of Berlin, Berlin, Germany; 3 Sanofi Genzyme, Bridgewater, NJ USA; 4 Present address: EMD Serono, Rockland, MA USA; 5 Regeneron Pharmaceuticals, Inc., Tarrytown, NY USA; 6 Present address: Intercept Pharmaceuticals, Inc., New York, NY USA; 7 University of Cantabria Hospital Universitario Marques de Valdecilla, Santander, Spain; 8 University of Copenhagen, Copenhagen, Denmark; 9 Metroplex Clinical Research Center and University of Texas Southwestern Medical Center, Dallas, TX USA

**Keywords:** Rheumatoid arthritis, Sarilumab, Adalimumab, Glycosylated haemoglobin, IL-6, Inflammation, Immuno-metabolism

## Abstract

**Background:**

Diabetes is common in patients with rheumatoid arthritis (RA). Interleukin (IL)-6 is implicated in both the pathogenesis of RA and in glucose homeostasis; this post hoc analysis investigated the effects of IL-6 receptor vs. tumour necrosis factor inhibition on glycosylated haemoglobin (HbA1c) in patients with RA with or without diabetes.

**Methods:**

Data were from two placebo-controlled phase III studies of subcutaneous sarilumab 150/200 mg q2w + methotrexate or conventional synthetic disease-modifying antirheumatic drugs (csDMARDs) and a phase III monotherapy study of sarilumab 200 mg q2w vs. adalimumab 40 mg q2w. Patients with diabetes were identified by medical history or use of antidiabetic medication (patients with HbA1c ≥ 9% were excluded from all three studies). HbA1c was measured at baseline and weeks 12/24. Safety and efficacy were assessed in RA patients with or without diabetes.

**Results:**

Patients with diabetes (*n* = 184) were older, weighed more and exhibited higher RA disease activity than patients without diabetes (*n* = 1928). Regardless of diabetes status, in patients on background csDMARDs, least squares (LS) mean difference (95% CI) in change from baseline in HbA1c for sarilumab 150 mg/200 mg vs. placebo at week 24 was − 0.28 (− 0.40, − 0.16; nominal *p* <  0.0001) and − 0.42 (− 0.54, − 0.31; nominal *p* <  0.0001), respectively. Without csDMARDs, LS mean difference for sarilumab 200 mg vs. adalimumab 40 mg at week 24 was − 0.13 (− 0.22, − 0.04; nominal *p* = 0.0043). Greater reduction in HbA1c than placebo or adalimumab was observed at week 24 with sarilumab in patients with diabetes and/or baseline HbA1c ≥ 7%. There was no correlation between baseline/change from baseline in HbA1c and baseline/change from baseline in C-reactive protein, 28-joint Disease Activity Score, or haemoglobin, nor between HbA1c change from baseline and baseline glucocorticoid use. Medical history of diabetes or use of diabetes treatments had limited impact on safety and efficacy of sarilumab and was consistent with overall phase III findings in patients with RA.

**Conclusions:**

In post hoc analyses, sarilumab was associated with a greater reduction in HbA1c than csDMARDs or adalimumab, independent of sarilumab anti-inflammatory effects. Prospective studies are required to further assess these preliminary findings.

**Trial registration:**

ClinTrials.gov NCT01061736: date of registration February 03, 2010; ClinTrials.gov NCT01709578: date of registration October 18, 2012; ClinTrials.gov NCT02332590: date of registration January 07, 2015.

## Introduction

The incidence of type 2 diabetes (T2D) is higher in patients with RA (17–20% [1–3]) than in the general population (8% [4]), independent of glucocorticoid use. Patients with RA also have increased insulin resistance compared with individuals without RA [3, 5]. RA disease outcomes are poorer in patients with comorbid diabetes, who are also at increased risk for cardiovascular disease relative to patients who have either RA or diabetes only [1, 2].

Chronic systemic inflammation is implicated in the pathogenesis of both RA and diabetes [6]. The pro-inflammatory cytokines interleukin (IL)-6, tumour necrosis factor-α (TNFα) and IL-1β play key roles in the synovial inflammation and joint damage associated with RA and also have systemic effects on extra-articular tissues [7–9]. IL-6 can signal through both membrane-bound (*cis*-signalling) and soluble (*trans*-signalling) IL-6 receptors (IL-6Rs) and therefore has pleiotropic effects on immune/inflammatory and other cell types, such as pancreatic β cells, skeletal muscle, adipose tissue and liver [10, 11]. Chronically elevated levels of systemic IL-6 have been associated with dysfunctional glucose metabolism and homeostasis and with the induction of insulin resistance in liver and adipose tissue [11–13]. Elevated levels of IL-6 are an independent risk factor for T2D [13–15], and IL-6 alone or in combination with IL-1β inhibits β cell function [16, 17]. Similarly, effects on glucose metabolism, insulin resistance, pancreatic β cell function and risk of diabetes are attributed to elevations in TNFα and IL-1β [6, 15, 18, 19], whilst IL-1β antagonism reduces hyperglycaemia and improves pancreatic β cell function in patients with T2D [20, 21]. Of note, the metabolic effect size of IL-1β antagonism is considerably greater in patients with RA and comorbid T2D [21], indicating that the efficacy of anticytokine biologics correlates with the inflammatory burden.

Medical management of T2D and RA can be complicated by the potential effects of RA treatments on glucose levels. Oral glucocorticoids increase the risk for diabetes in patients with RA [22, 23] because of the adverse metabolic actions of these drugs, with higher dose and longer treatment duration increasing the risk [23]. By contrast, the anti-inflammatory drug hydroxychloroquine reduced the risk of incident diabetes in patients with RA [24, 25] and was also associated with a favourable effect on glycaemia in patients with RA in the absence of diabetes [26]. These effects are not necessarily associated with direct actions on insulin resistance and/or pancreatic β cell function [24, 25, 27], but rather to a reduction of low-grade inflammation by inhibiting the inflammasome [28]. Methotrexate (MTX) also reduces glycosylated haemoglobin (HbA1c) levels and the risk for diabetes in patients with RA [29], likely independently of insulin sensitivity [30].

Because diabetes is a common comorbidity in patients with RA and cytokines are implicated in glucose homeostasis, we conducted post hoc analyses of three sarilumab phase III randomised clinical trials. These analyses aimed to assess the effect of sarilumab (a human monoclonal antibody that blocks the IL-6Rα), as monotherapy or in combination with conventional synthetic disease-modifying antirheumatic drugs (csDMARDs) on HbA1c levels compared with either placebo (+ MTX/csDMARD) or adalimumab monotherapy [31–33]. We also assessed the safety and efficacy of sarilumab in patients with RA with or without comorbid diabetes.

## Methods

### Study design

Details of the three phase III study designs have been described previously [31–33]. In brief, the MOBILITY trial (NCT01061736) was conducted to investigate the efficacy and safety of up to 52 weeks of sarilumab (or placebo) in combination with MTX in patients with moderate-to-severe active RA and an inadequate response to MTX (MTX-IR); this trial is referred to herein as the MTX-IR sarilumab + MTX study. The TARGET trial (NCT01709578) was conducted to investigate the efficacy and safety of up to 24 weeks of sarilumab (or placebo) in combination with background conventional synthetic DMARDs (csDMARDs) in patients with moderate-to-severe RA who were intolerant of, or who had inadequate response to, TNF inhibitors (TNFi-INT/IR); this study is referred to herein as the TNFi-INT/IR sarilumab + csDMARDs study. The MONARCH trial (NCT02332590) was conducted to compare the 24-week efficacy and safety of sarilumab monotherapy with adalimumab monotherapy in biologic DMARD-naïve patients with moderate-to-severe active RA who were intolerant of, or had an IR to, MTX (MTX-INT/IR); this study is referred to herein as the monotherapy study. Patients with uncontrolled diabetes mellitus (defined by HbA1c ≥ 9% at the screening visit) were excluded from participation. Patients treated with ≤ 10 mg oral prednisone or equivalent at a stable dose for at least 4 weeks prior to the baseline visit were included; however, changes in dose were not permitted during the double-blind treatment periods unless required for treatment of an adverse event (AE) (other than worsening RA).

The three protocols were approved by the appropriate ethics committees/institutional review boards, and each patient provided written informed consent before participation in the study. The studies were conducted in compliance with institutional review board regulations, the International Conference on Harmonisation Guidelines for Good Clinical Practice and the Declaration of Helsinki.

For the purposes of these post hoc analyses, patients were classified as having diabetes if they reported either a medical history of the disease or baseline use of medication to treat diabetes (e.g. metformin, sulfonylureas, dipeptidyl peptidase 4 inhibitors or insulins).

### Assessments

HbA1c was measured at baseline in all three studies and also at weeks 12 and 24 in the TNFi-INT/IR sarilumab + csDMARD and monotherapy studies. In the MTX-IR sarilumab + MTX study, HbA1c was assessed after baseline at the investigators’ discretion.

Safety assessments included the incidence of treatment-emergent adverse events (TEAEs), serious TEAEs (SAEs), serious infections and specific abnormalities in laboratory tests. AEs were described at the Medical Dictionary for Regulatory Activities (version 16.0) preferred-term level.

Efficacy assessments included the American College of Rheumatology 20% (ACR20) response rate and the change from baseline in the Health Assessment Questionnaire-Disability Index (HAQ-DI), Disease Activity Score (28 joints) using C-reactive protein (DAS28-CRP) and Clinical Disease Activity Index (CDAI) at week 24.

### Statistical methods

The analyses of patients with and without diabetes were conducted post hoc; therefore, *p* values should be considered nominal.

Changes in HbA1c were analysed for subgroups defined by a medical history of diabetes or baseline use of an antidiabetic medication. To determine whether changes in HbA1c might be therapeutically relevant for patients with marginal glycaemic control, the analyses were repeated in a subgroup of patients who had a baseline HbA1c value ≥ 7.0% [34]. To investigate potential modulators of HbA1c, other than the investigational treatment, patients were also classified by oral glucocorticoid use and type of treatment for diabetes, and Spearman’s rank correlation coefficients (*r*_S_) were calculated for baseline HbA1c vs. baseline CRP, DAS28-CRP and haemoglobin and for changes in HbA1c vs. changes in CRP, DAS28-CRP and haemoglobin.

Values for observed cases were used without imputation for analyses of safety and laboratory parameters. No formal statistical analysis of AEs was conducted for patients with RA in the presence vs. absence of diabetes (comparisons were descriptive).

Efficacy was assessed in the intent-to-treat population. For efficacy analysis, data from the MTX-IR sarilumab + MTX study and the TNFi-INT/IR sarilumab + csDMARD studies were pooled. For categorical efficacy variables, patients were considered nonresponders from the time they started rescue therapy or discontinued the study medication. For continuous efficacy variables, assessments were set to missing from the time a patient received rescue therapy or discontinued study medication before the end of the study. Missing values were not imputed. Changes in efficacy variables were modelled using a repeated-measures-mixed-effect model, assuming unstructured covariance with treatment, region, visit, subgroup, treatment-by-visit interaction, treatment-by-subgroup interaction and treatment-by-visit-by-subgroup interaction included in the model. A nominal *p* value < 0.05 was considered significant.

## Results

### Patient baseline characteristics and disposition

Across the three phase III studies, 184 of 2112 (8.7%) patients had a medical history of diabetes or concomitant use of antidiabetic treatment at baseline (MTX-IR sarilumab + MTX study: *n* = 91; TNFi-INT/IR sarilumab + csDMARDs study: *n* = 67; monotherapy study: *n* = 26) (Table 1). The majority of patients with diabetes in each study (75–82%) were receiving concomitant antidiabetic medications at baseline, with noninsulin blood glucose-lowering medications being the most common (Table 1).

**Table 1:** Baseline characteristics of patients with and without diabetes (medical history of diabetes or concomitant use of antidiabetic treatment) across three phase III studies

	**With diabetes (*N* = 184)**	**Without diabetes (*N* = 1928)**	***p*** **value**
Age (years)			< 0.0001
Mean (SD)	56.5 (9.4)	51.0 (12.1)	
Sex, *n* (%)			0.3348
Female	146 (79.3)	1585 (82.2)	
Male	38 (20.7)	343 (17.8)	
Race, *n* (%)			0.0907
Caucasian/White	147 (79.9)	1607 (83.4)	
Black	8 (4.3)	44 (2.3)	
Asian	15 (8.2)	99 (5.1)	
Other	14 (7.6)	178 (9.2)	
Weight (kg)			< 0.0001
Mean (SD)	84.08 (21.59)	74.04 (18.76)	
Body mass index (kg/m^2^)			< 0.0001
Mean (SD)	32.18 (7.40)	28.04 (6.38)	
Duration of RA since diagnosis (years)			0.6294
Mean (SD)	9.82 (8.81)	9.51 (8.44)	
Rheumatoid factor, *n* (%)			0.0964
Positive	136 (74.3)	1517 (79.5)	
Negative	47 (25.7)	390 (20.5)	
Anti CCP antibody, *n* (%)			0.0593
Positive	143 (77.7)	1587 (83.2)	
Negative	41 (22.3)	320 (16.8)	
Tender joint count (0–68)			0.0017
Mean (SD)	30.97 (15.92)	27.11 (14.11)	
Swollen joint count (0–66)			0.0185
Mean (SD)	19.72 (11.90)	17.57 (10.02)	
HAQ-DI (0–3)			0.0010
Mean (SD)	1.82 (0.66)	1.66 (0.63)	
CRP (mg/L)			0.3210
Mean (SD)	24.90 (29.39)	22.67 (24.22)	
Oral glucocorticosteroid use, *n* (%)	119 (64.7)	1177 (61.0)	0.3344
Mean (SD) prednisone equivalent dose, mg	6.6 (2.63)	6.6 (2.68)	0.7462
Hydroxychloroquine use, *n* (%)	4 (2.2)	33 (1.7)	> 0.9999
Antidiabetic medication use, *n* (%)	143 (77.7)	NR	NR
Any noninsulin blood glucose-lowering drug, *n* (%)^a^	85 (46.2)	NR	NR
≥ 2 noninsulin blood glucose-lowering drugs, *n* (%)^b^	32 (17.4)	NR	NR
Any insulin and analogue without oral blood glucose lowering drug, *n* (%)	13 (7.1)	NR	NR
Any insulin and analogue plus oral blood glucose lowering drug, *n* (%)	14 (7.6)	NR	NR

Percentages are calculated using number of patients assessed as denominator

*p* values are based on *t* test for continuous variables and chi-square test if frequency in all cells ≥ 5 and Fisher exact test if frequency in at least one cell < 5, for categorical variables

*CCP* cyclic citrullinated peptide, *CRP* C-reactive protein, *HAQ-DI* Health Assessment Questionnaire-Disability Index, *Number* number of patients assessed, *NR* not relevant, *RA* rheumatoid arthritis, *SD* standard deviation

^a^Excluding insulins, monotherapy

^b^Excluding insulins

Patients with diabetes were significantly older (56.5 years vs. 51.0 years; *p* <  0.0001), weighed more (84.1 kg vs. 74.0 kg; *p* <  0.0001) and had a significantly higher body mass index (32.2 kg/m^2^ vs. 28.0 kg/m^2^; *p* <  0.0001) at baseline than patients without diabetes. Patients with diabetes also had a significantly higher mean swollen joint count and tender joint count and greater disability at baseline than those without diabetes (Table 1). The proportion of patients with or without diabetes who were receiving glucocorticoids (≤ 10 mg prednisone or equivalent) at baseline was 69.2% and 62.6%, respectively, in the MTX-IR sarilumab + MTX study, 59.7% and 62.8%, respectively, in the TNFi-INT/IR sarilumab + csDMARDs study and 65.4% and 53.9%, respectively, in the monotherapy study.

Rates of study completion were similar between patients with and without diabetes (Table S1).

### Reduction in HbA1c

**Fig. 1:**
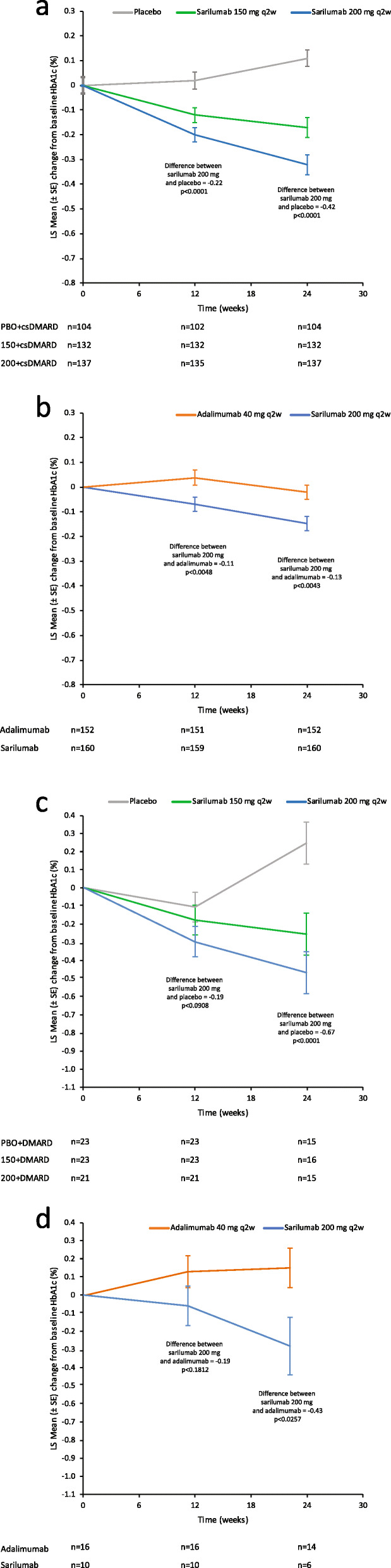
Change in HbA1c in patients with RA (irrespective of diabetes status) treated with **a** sarilumab 150/200 mg q2w + csDMARDs or placebo + csDMARDs and **b** sarilumab 200 mg q2w or adalimumab 40 mg q2w. Change in HbA1c in patients with RA and diabetes treated with **c** sarilumab 150/200 mg q2w + csDMARDs or placebo + csDMARDs and **d** sarilumab 200 mg q2w or adalimumab 40 mg q2w. *p* values are nominal. *csDMARD*, conventional disease-modifying antirheumatic drug; *HbA1c*, glycosylated haemoglobin; *LS*, least squares; *PBO*, placebo; *q2w*, every 2 weeks; *RA*, rheumatoid arthritis; *SE*, standard error

Patients with RA who were TNFi-INT/IR (irrespective of diabetic status) and were treated with sarilumab + csDMARDs exhibited a greater reduction in HbA1c from baseline to week 24 compared with those treated with placebo + csDMARDs (Fig. 1a). The least squares (LS) mean differences (95% CI; nominal *P*) for sarilumab 150 mg q2w + csDMARD and sarilumab 200 mg q2w + csDMARDs, respectively, vs. placebo + csDMARDs were − 0.28 (− 0.40, − 0.16; *p* <  0.0001) and − 0.42 (− 0.54, − 0.31; *p* < 0.0001). In the monotherapy study, patients with RA who were MTX-INT/IR and treated with sarilumab also exhibited a greater reduction in HbA1c than those treated with adalimumab (Fig. 1b). Notably, the LS mean difference (95% CI; nominal *p*) for sarilumab 200 mg q2w from adalimumab 40 mg q2w at week 24 was − 0.13 (− 0.22, − 0.04; *p* = 0.0043). In all three studies, reductions in HbAc1 with sarilumab occurred in patients who achieved disease activity thresholds of DAS28-CRP < 2.6 or < 3.2 (low disease activity) as well as in patients who did not achieve disease activity thresholds (Table S2).

For patients with a medical history of diabetes or treatment for diabetes, the reduction in HbA1c at week 24 (Fig. 1c, d) was greater with sarilumab + csDMARDs (LS mean difference, − 0.67 [− 0.98, − 0.37; *p* < 0.0001] and − 0.47 [− 0.77, − 0.17; *p* = 0.0021] for the sarilumab 200 mg q2w group and 150 mg q2w group, respectively) than for patients treated with placebo + csDMARDs. In the monotherapy study, the reduction in HbA1c was greater with sarilumab 200 mg q2w than with adalimumab 40 mg q2w (LS mean difference, − 0.43 [− 0.80, − 0.05; *p* = 0.0257]).

Among patients with baseline HbA1c greater than the target of 7.0%, those who received sarilumab + csDMARDs had a greater reduction from baseline in HbA1c at week 24 compared with those who received placebo + csDMARDs (*p* = 0.0097; *p* = 0.0003). A similar result was seen in those who received sarilumab compared with adalimumab monotherapy (*p* = 0.0002; Table 2).

**Table 2:** Change from baseline at week 24 in HbA1c in patients with RA and subgroups of patients with RA and diabetes from phase III studies of sarilumab + csDMARDs or sarilumab monotherapy

	**Combination therapy with csDMARDs in TNFi-INT/IR patients**			**Monotherapy in MTX-INT/IR patients**	
**Change in HbA1c at week 24**	**Placebo + csDMARDs**	**Sarilumab 150 mg q2w + csDMARDs**	**Sarilumab 200 mg q2w + csDMARDs**	**Adalimumab 40 mg q2w monotherapy**	**Sarilumab 200 mg q2w monotherapy**
**Patients with a medical history of diabetes or baseline use of antidiabetic medication**					
Number	15	16	15	14	6
Baseline mean (SD)	7.20 (1.06)	6.94 (1.08)	6.96 (1.09)	6.77 (0.89)	6.65 (1.17)
LS mean change (SE)^a^	0.23 (0.11)	− 0.24 (0.11)	− 0.44 (0.11)	0.15 (0.11)	− 0.28 (0.16)
LS mean difference, 95% CI^a^		− 0.47 (− 0.77, − 0.17)	− 0.67 (− 0.98, − 0.37)		− 0.43 (− 0.80, − 0.05)
*p* value vs. placebo/adalimumab^a^		0.0021	< 0.0001		0.0257
**Baseline HbA1c ≥ 7.0%**					
Number	11	10	9	6	4
Baseline mean (SD)	7.80 (0.58)	7.77 (0.59)	7.88 (0.61)	7.57 (0.49)	7.78 (0.54)
LS mean change (SE)^a^	− 0.08 (0.13)	− 0.56 (0.13)	− 0.77 (0.14)	0.29 (0.16)	− 0.67 (0.20)
LS mean diff, 95% CI^a^		− 0.48 (− 0.84, − 0.12)	− 0.69 (− 1.06, − 0.32)		− 0.96 (− 1.46, − 0.46)
*p* value vs. placebo/adalimumab^a^		0.0097	0.0003		0.0002

All assessments are set to missing from the time a patient prematurely discontinues study medication

*CI* confidence interval, *csDMARD* conventional synthetic disease-modifying antirheumatic drug, *HbA1c* glycosylated haemoglobin, *INT* intolerant, *IR* inadequate response, *LS* least squares, *MMRM* mixed-effect model repeat measurement, *MTX* methotrexate, *Number* number of patients with assessment at both baseline and week 24, *q2w* every 2 weeks, *SD* standard deviation, *SE* standard error, *TNFi* tumour necrosis factor-α inhibitor

^a^MMRM assuming an unstructured covariance structure. Model = subgroup, treatment, region, visit, treatment-by-visit interaction, treatment-by-subgroup interaction and treatment-by-visit-by-subgroup interaction. Nominal *p* values for differences between sarilumab and comparator

Baseline HbA1c values did not correlate with baseline CRP, DAS28-CRP or haemoglobin levels in the overall study population in any of the three studies (all *r*_S_ < 0.1), even when patients with a baseline HbA1c value ≥ 7% were included. There was no interaction between the reduction in HbA1c levels at week 12 or 24 and baseline glucocorticoid use or the increase in haemoglobin levels in patients with RA in the absence or presence of diabetes (Table S3). In the TNFi-INT/IR sarilumab + csDMARDs study, changes in HbA1c at week 12 or 24 did not correlate with change in CRP (all *r*_S_ < 0.16), DAS28-CRP (all *r*_S_ < 0.1) or haemoglobin (all *r*_S_ < 0.1). In the monotherapy study, a weak positive correlation (*r*_S_ < 0.24) was observed between changes in HbA1c and changes in CRP at week 24 in both the sarilumab 200 mg q2w (*r*_S_ 0.236; *p* = 0.003) and adalimumab 40 mg q2w groups (*r*_S_ 0.174; *p* = 0.035) and the change in HbA1c and DAS28-CRP in the adalimumab group (0.188; *p* = 0.023). In patients who received sarilumab + csDMARDs or sarilumab monotherapy, changes in HbA1c at week 24 were similar whether or not increases in haemoglobin levels were observed at week 12 (data not shown) and week 24 (Table S3). Similar results were observed in models adjusted for various measures of disease activity (Table S4).

### Rheumatoid arthritis disease activity efficacy outcomes

Efficacy outcomes at week 24 for ACR20 and change from baseline in HAQ-DI, DAS28-CRP and CDAI are shown in Fig. S1. In the pooled analysis of the two placebo-controlled studies of sarilumab + MTX/csDMARDs, treatment by diabetes status interaction tests were not significant for ACR20 (*p* = 0.224) or for change from baseline in HAQ-DI (*p* = 0.475), DAS28-CRP (*p* = 0.110) or CDAI (*p* = 0.597). In the monotherapy trial, the small number of patients with diabetes limited data interpretation; however, treatment by subgroup interaction test results were not significant for ACR20 (*p* = 0.585) or for change from baseline in HAQ-DI (*p* = 0.719) but were significant for change from baseline in DAS28-CRP (*p* = 0.003) and CDAI (*p* = 0.025).

### Safety

The rates of treatment-emergent SAEs and TEAEs leading to death or treatment discontinuation during the double-blind treatment phase in the three studies are shown for patients with or without diabetes in Table 3. In the placebo-controlled studies, the overall TEAE rate for most treatment groups was numerically higher in patients with diabetes compared with those without diabetes (with sarilumab 200 mg + MTX: 97.3% vs. 76.9%; placebo + MTX: 74.1% vs. 61.1%; with sarilumab 200 mg + csDMARDs: 61.9% vs. 65.6%; and with placebo + csDMARDs: 73.9% vs. 46.2%). Of note, in the monotherapy trial, TEAE rates were similar for patients treated with sarilumab vs. adalimumab, and within treatment groups the TEAE rate was numerically higher for patients with diabetes than for those without (sarilumab: 70.0% vs. 63.8%; adalimumab: 68.8% vs. 63.1%, respectively; Table 3). However, the total number of patients with diabetes in each treatment group in the monotherapy trial was low compared with the number of patients without diabetes (Table 3), which limited comparisons.

**Table 3:** Overview of TEAEs during the double-blind treatment phases of phase III trials

	**Combination therapy with MTX in MTX-IR patients**			**Combination therapy with csDMARDs in TNFi-INT/IR patients**			**Monotherapy in MTX-INT/IR patients**		
	**52 weeks**			**24 weeks**			**24 weeks**		
	**Placebo**	**Sarilumab 150 mg q2w**	**Sarilumab 200 mg q2w**	**Placebo**	**Sarilumab 150 mg q2w**	**Sarilumab 200 mg q2w**	**Adalimumab 40 mg q2w**	**Sarilumab 200 mg q2w**	**Total**
**TEAE,** ***n*/*N*** ** (%)**									
With diabetes	20/27 (74.1)	21/27 (77.8)	36/37 (97.3)	17/23 (73.9)	18/23 (78.3)	13/21 (61.9)	11/16 (68.8)	7/10 (70.0)	143/184 (77.7)
Without diabetes	226/370 (61.1)	279/374 (74.6)	276/359 (76.9)	73/158 (46.2)	101/158 (63.9)	107/163 (65.6)	106/168 (63.1)	111/174 (63.8)	1279/1924 (66.5)
**Treatment-emergent SAE,** ***n*/*N*** ** (%)**									
With diabetes	4/27 (14.8)	3/27 (11.1)	5/37 (13.5)	1/23 (4.3)	2/23 (8.7)	2/21 (9.5)	1/16 (6.3)	0/10	18/184 (9.8)
Without diabetes	17/370 (4.6)	34/374 (9.1)	40/359 (11.1)	5/158 (3.2)	4/158 (2.5)	8/163 (4.9)	11/168 (6.5)	9/174 (5.2)	128/1924 (6.7)
**TEAE leading to death,** ***n*/*N*** ** (%)**									
With diabetes	1/27 (3.7)	0/27	0/37	0/23	0/23	0/21	0/16	0/10	1/184 (0.5)
Without diabetes	1/370 (0.3)	2/374 (0.5)	1/359 (0.3)	1/158 (0.6)	0/158	0/163	0/168	1/174 (0.6)	6/1924 (0.3)
**TEAE leading to treatment discontinuation,** ***n*/*N*** ** (%)**									
With diabetes	2/27 (7.4)	1/27 (3.7)	5/37 (13.5)	1/23 (4.3)	3/23 (13.0)	2/21 (9.5)	2/16 (12.5)	1/10 (10.0)	17/184 (9.2)
Without diabetes	18/370 (4.9)	50/374 (13.4)	49/359 (13.6)	7/158 (4.4)	11/158 (7.0)	15/163 (9.2)	11/168 (6.5)	10/174 (5.7)	171/1924 (8.9)

*csDMARD* conventional synthetic disease-modifying antirheumatic drug, *INT* intolerant, *IR* inadequate response, *MTX* methotrexate, *q2w* every 2 weeks, *SAE* serious TEAE, *TEAE* treatment-emergent adverse event, *TNFi* tumour necrosis factor-α inhibitor

Rates of infection were higher in patients with diabetes than without diabetes for all treatment groups, except for patients who were TNFi-INT/IR treated with sarilumab 200 mg q2w + csDMARDs (Table 4). The rate of serious infections, opportunistic infections and infections leading to treatment discontinuation was low and comparable in patients with or without diabetes who were treated with sarilumab. There were no cases of tuberculosis among patients with diabetes; one case of tuberculosis was reported in a patient without diabetes who received adalimumab monotherapy (Table 4).

**Table 4:** Selected treatment-emergent adverse events

	**Combination therapy with MTX in MTX-IR patients** **52 weeks**			**Combination therapy with csDMARDs in TNFi-INT/IR patients** **24 weeks**			**Monotherapy in MTX-INT/IR patients** **24 weeks**		**Total**
	**Placebo + MTX**	**Sarilumab** **150 mg q2w**	**Sarilumab** **200 mg q2w**	**Placebo**	**Sarilumab** **150 mg q2w**	**Sarilumab** **200 mg q2w**	**Adalimumab** **40 mg q2w**	**Sarilumab** **200 mg q2w**	
**Patients with ≥ 1 infection,** ***n*/*N*** ** (%)**									
With diabetes	12/27 (44.4)	12/27 (44.4)	20/37 (54.1)	8/23 (34.8)	10/23 (43.5)	4/21 (19.0)	9/16 (56.3)	3/10 (30.0)	78/184 (42.4)
Without diabetes	115/370 (31.1)	156/374 (41.7)	139/359 (38.7)	40/158 (25.3)	30/158 (19.0)	52/163 (31.9)	42/168 (25.0)	50/174 (28.7)	624/1924 (32.4)
**Patients with ≥ 1 serious infection,** ***n*/*N*** ** (%)**									
With diabetes	4/27 (14.8)	1/27 (3.7)	1/37 (2.7)	0/23	1/23 (4.3)	0/21	0/16	0/10	7/184 (3.8)
Without diabetes	6/370 (1.6)	10/374 (2.7)	14/359 (3.9)	2/158 (1.3)	0/158	2/163 (1.2)	2/168 (1.2)	2/174 (1.1)	38/1924 (2.0)
**Patients with infection leading to treatment discontinuation,** ***n*/*N*** ** (%)**									
With diabetes	1/27 (3.7)	0/27	0/37	0/23	3/23 (13.0)	0/21	0/16	0/10	4/184 (2.2)
Without diabetes	5/370 (1.4)	13/374 (3.5)	11/359 (3.1)	1/158 (0.6)	2/158 (1.3)	5/163 (3.1)	2/168 (1.2)	1/174 (0.6)	40/1924 (2.1)
**Patients with opportunistic infections (including tuberculosis),** ***n*/*N*** ** (%)**									
With diabetes	0/27	0/27	1/37 (2.7)	0/23	0/23	0/21	0/16	0/10	1/184 (0.5)
Without diabetes	2/370 (0.5)	2/374 (0.5)	3/359 (0.8)	1/158 (0.6)	0/158	2/163 (1.2)	1/168 (0.6)	1/174 (0.6)	12/1924 (0.6)
**Patients with tuberculosis,** ***n*/*N*** ** (%)**									
With diabetes	0/27	0/27	0/37	0/23	0/23	0/21	0/16	0/10	0/184
Without diabetes	0/370	0/374	0/359	0/158	0/158	0/163	1/168 (0.6)	0/174	1/1924 (0.1)
**Patients with ≥ 1 hypersensitivity,** ***n*/*N*** ** (%)**									
With diabetes	1/27 (3.7)	1/27 (3.7)	3/37 (8.1)	2/23 (8.7)	3/23 (13.0)	4/21 (19.0)	0/16	2/10 (20.0)	16/184 (8.7)
Without diabetes	17/370 (4.6)	25/374 (6.7)	26/359 (7.2)	5/158 (3.2)	7/158 (4.4)	7/163 (4.3)	10/168 (6.0)	8/174 (4.6)	105/1924 (5.5)
**Patients with ≥ 1 serious hypersensitivity,** ***n*/*N*** ** (%)**									
With diabetes	0/27	0/27	0/37	0/23	0/23	0/21	0/16	0/10	0/184
Without diabetes	0/370	0/374	1/359 (0.3)	0/158	0/158	0/163	1/168 (0.6)	0/174	2/1924 (0.1)
**Patients with ≥ 1 hypersensitivity leading to treatment discontinuation,** ***n*/*N*** ** (%)**									
With diabetes	0/27	0/27	1/37 (2.7)	0/23	0/23	0/21	0/16	1/10 (10.0)	2/184 (1.1)
Without diabetes	1/370 (0.3)	3/374 (0.8)	1/359 (0.3)	0/158	0/158	1/163 (0.6)	1/168 (0.6)	0/174	7/1924 (0.4)
**ANC < 1.5 G/L (non-black patients) or < 1.0 G/L (black patients),** ***n*/*N*** ** (%)**									
With diabetes	1/27 (3.7)	1/27 (3.7)	5/37 (13.5)	0/23	1/23 (4.3)	5/21 (23.8)	1/16 (6.3)	3/10 (30.0)	17/184 (9.2)
Without diabetes	5/370 (1.4)	72/374 (19.3)	88/359 (24.5)	2/158 (1.3)	30/158 (19.0)	37/162 (22.8)	39/167 (23.4)	59/174 (33.9)	332/1922 (17.3)
**Platelets < 50 G/L,** ***n*/*N*** ** (%)**									
With diabetes	0/27	0/27	0/37	0/23	0/23	0/21	0/16	0/10	0/184
Without diabetes	0/370	0/374	2/358 (0.6)	0/158	0/158	1/162 (0.6)	0/0	1/174 (0.6)	4/1921 (0.2)
**Total cholesterol baseline normal/missing to post baseline ≥ 6.2 mmol/L,** ***n*/*N*** ** (%)**									
With diabetes	6/25 (24.0)	2/23 (8.7)	12/30 (40.0)	6/19 (31.6)	9/23 (39.1)	7/20 (35.0)	2/15 (13.3)	2/7 (28.6)	46/162 (28.4)
Without diabetes	62/325 (19.1)	128/327 (39.1)	140/315 (44.4)	18/140 (12.9)	51/130 (39.2)	52/141 (36.9)	35/131 (26.7)	53/140 (37.9)	539/1649 (32.7)
**Total LDL-C baseline normal/missing to post-baseline LDL-C ≥ 4.1 mmol/L,** ***n*/*N*** ** (%)**									
With diabetes	2/25 (8.0)	3/25 (12.0)	11/32 (34.4)	4/21 (19.0)	7/22 (31.8)	5/20 (25.0)	1/15 (6.7)	2/9 (22.2)	35/169 (20.7)
Without diabetes	41/344 (11.9)	82/342 (24.0)	101/334 (30.2)	10/148 (6.8)	39/146 (26.7)	38/151 (25.2)	28/142 (19.7)	40/153 (26.1)	379/1760 (21.5)
**Total triglycerides baseline normal/missing to post-baseline ≥ 4.6 mmol/L,** ***n*/*N*** ** (%)**									
With diabetes	2/27 (7.4)	0/0	3/36 (8.3)	0/23	1/23 (4.3)	0/21	1/16 (6.3)	1/10 (10.0)	8/181 (4.4)
Without diabetes	1/368 (0.3)	11/372 (3.0)	13/358 (3.6)	1/157 (0.6)	5/153 (3.3)	6/161 (3.7)	4/164 (2.4)	3/173 (1.7)	44/1906 (2.3)
**ALT** > **3 to ≤ 5 ULN,** ***n*/*N*** ** (%)**									
With diabetes	0/27	0/27	6/37 (16.2)	0/23	0/23	0/21	2/16 (12.5)	0/10	8/184 (4.3)
Without diabetes	9/370 (2.4)	33/374 (8.8)	25/359 (7.0)	2/158 (1.3)	4/157 (2.5)	7/161 (4.3)	10/167 (6.0)	11/174 (6.3)	101/1920 (5.3)
**ALT** > **5 to ≤ 10 ULN,** ***n*/*N*** ** (%)**									
With diabetes	0/27	0/27	2/37 (5.4)	0/23	0/23	0/21	1/16 (6.3)	0/10	3/184 (1.6)
Without diabetes	1/370 (0.3)	11/374 (2.9)	8/359 (2.2)	0/158	0/158	0/161	2/167 (1.2)	3/174 (1.7)	25/1920 (1.3)

*ALT* alanine aminotransferase, *ANC* absolute neutrophil count, *csDMARD* conventional synthetic disease-modifying antirheumatic drug, *INT* intolerant, *IR* inadequate response, *LDL-C* low-density lipoprotein cholesterol, *MTX* methotrexate, *q2w* every 2 weeks, *TNFi* tumour necrosis factor α inhibitor, *ULN* upper limit of normal

The overall incidence of hypersensitivity reactions was numerically greater among patients with diabetes (Table 4); however, no patients with diabetes experienced serious hypersensitivity reactions. Two patients without diabetes who were treated with sarilumab + csDMARDs (*n* = 1) or adalimumab monotherapy (*n* = 1) experienced serious hypersensitivity reactions. Regarding hypersensitivity reactions leading to treatment discontinuation: these were reported in two patients with diabetes who were treated with sarilumab 200 mg + csDMARD (*n* = 1) or monotherapy (*n* = 1) and five patients without diabetes who were treated with sarilumab 150 mg q2w + MTX (*n* = 3), sarilumab 200 mg q2w + MTX (*n* = 1) or sarilumab 200 mg q2w + csDMARDs (*n* = 1).

The incidence of selected laboratory abnormalities is shown in Table 4. No spontaneously reported symptomatic or biochemical hypoglycaemia TEAEs were reported in any of the three studies. A reduced absolute neutrophil count (ANC) was generally seen more frequently in patients without diabetes across all studies. Platelet counts < 50 G/L were uncommon in both groups and not observed in the 184 patients with diabetes. Alanine aminotransferase elevations were not increased in patients with diabetes compared with those without diabetes. The proportions of patients with shifts from baseline to highest post-baseline cholesterol (≥ 6.2 mmol/L [≥ 239.7 mg/dL]), low-density lipoprotein cholesterol (≥ 4.1 mmol/L [≥ 158.5 mg/dL]) and triglycerides (≥ 4.6 mmol/L [≥ 407.1 mg/dL]) were not consistently higher in patients with diabetes compared with those without diabetes (Table 4).

There were no interactions between diabetes status and incidence of weight gain > 5%. Among patients treated with sarilumab 200 mg q2w, the percentages of patients with > 5% weight gain at week 24 with and without diabetes were 17.2% vs. 18.4%, 13.3% vs. 18.3% and 14.3% vs. 11.9% for patients treated with sarilumab + MTX, sarilumab + csDMARDs or sarilumab monotherapy, respectively (Table S5).

## Discussion

Diabetes is a common comorbid condition among patients with RA [1, 2, 35]. Medical management of both conditions can be complicated, because RA disease activity and the use of glucocorticoids, csDMARDs and bDMARDs (often in combination) to treat RA may affect glucose levels through their effects on glucose metabolism, insulin sensitivity and pancreatic β cell function [3, 24, 25, 36].

These post hoc analyses of three phase III clinical studies in patients with RA show that sarilumab as monotherapy or in combination with MTX/csDMARDs is associated with a greater reduction in HbA1c than adalimumab monotherapy or placebo + MTX/csDMARDs, particularly in patients with diabetes. Reductions in HbA1c were more prominent in patients treated with sarilumab compared with either MTX/csDMARDs or adalimumab in patients whose baseline HbA1c was ≥ 7%, a level that exceeds the target HbA1c of < 7% recommended by the American Diabetes Association, supporting the possibility of improving, as well as maintaining, glucose homeostasis.

It is well recognised that oral glucocorticoids, in addition to immune-suppressive actions, also affect glucose homeostasis. In RA, oral glucocorticoids are commonly used and it is important to notice that the effects of sarilumab on HbA1c were not attenuated by concomitant glucocorticoid treatment (≤ 10 mg prednisone equivalent). In addition, the observed reduction in HbA1c could not be explained by the expected increase in haemoglobin associated with IL-6R blockade, which indicates that its effect on HbA1c did not reflect an indirect effect of a change in haemoglobin levels. Whilst the current analyses cannot exclude the general influence of systemic inflammation as a cause of changes in glycaemia, a correlation between changes in HbA1c and changes in CRP was not observed in the TNFi-INT/IR sarilumab + csDMARDs study and correlations were minimal when sarilumab was used as monotherapy (Spearman’s rank correlations < 0.24). Similar results were observed with three models incorporating different measures of change in disease activity and inflammation (CRP and CDAI; DAS28-CRP; and tender and swollen 28 joint counts, patient and physician global assessments, and CRP). These results support an effect of sarilumab in reducing HbA1c that is independent of anti-inflammatory effects, although some degree of association between general effects on inflammation and changes in glycaemia cannot be ruled out.

These analyses also showed that sarilumab + MTX/csDMARDs or as monotherapy is efficacious in patients with or without diabetes; this is consistent with the overall efficacy findings from all three phase III studies [31–33]. Furthermore, there were no major differences in the safety profile of sarilumab in patients with RA in the presence or absence of diabetes, although patients with uncontrolled diabetes and therefore potentially greater risk were excluded from these studies. These findings are reassuring given the possible vulnerabilities associated with diabetes (e.g. patients with poor glycaemic control have greater susceptibility to developing infections, especially if they are older or more likely to be receiving oral glucocorticoids [37]). In this analysis, the rate of serious infections, opportunistic infections and infections leading to treatment discontinuation was similar in patients with or without diabetes, despite a numerically higher rate of decreased ANC in patients with diabetes. Patients with treated diabetes are also at risk for hypoglycaemia, especially those whose treatment includes insulin or an insulin secretagogue [38]. There were no reports of symptomatic or biochemical hypoglycaemia in any of the studies, even though approximately 17% of the patients with diabetes were taking ≥ 2 noninsulin blood glucose-lowering medications and approximately 8% were using insulin or an insulin secretagogue. Modelling to assess the effect of concomitant use of hydroxychloroquine, known to induce symptomatic hypoglycaemia, by diabetic status showed no significant interaction at week 12 or 24 (data not shown). Changes in lipid parameters were generally comparable in the presence or absence of diabetes, and no interaction between diabetes and weight gain was observed. This finding may be particularly important, given the association between RA and comorbid diabetes and cardiovascular risk [39].

These analyses have some notable limitations: they were conducted post hoc; and none of the studies included were designed specifically to evaluate HbA1c levels. The patients who had diabetes were selected on the basis of previous clinical history or current antidiabetic medication use, and patients with uncontrolled diabetes, defined as HbA1c ≥ 9.0%, were excluded. Whilst this exclusion is standard in trials, it does introduce a bias in the study population when compared with the general population. In addition, diet and exercise were not monitored systematically during the study, and there were no specific recommendations to maintain dietary or exercise habits in the individual study protocols. In the monotherapy study, the number of patients with diabetes in the two treatment groups was small. The proportion of non-white patients, in whom the prevalence of diabetes may differ, was low in the pooled studies. No analyses were performed to investigate any differences between such populations. Despite these limitations, findings across the three studies were consistent.

Although the analyses were conducted post hoc, data were collected in a prospective and blinded manner, and blood samples were analysed by a central laboratory, reducing assay variability. Although the numbers of diabetic patients in the sarilumab studies were small, they were larger than those reporting reductions in HbA1c in previous studies of tocilizumab, in which patients were treated openly (*n* = 10 [40] and *n* = 34 [41]) or only evaluated HbA1c after 104 weeks [42]. In none of the aforementioned tocilizumab studies nor during the course of randomised, well-controlled, multinational studies designed to support marketing authorisation of a therapy for RA was HbA1c collected, nor were patients as thoroughly characterised with respect to concomitant glucocorticoid use, treatment for diabetes, or safety of the treatments. Other studies of the effects of IL-6 receptor blockade on insulin sensitivity/resistance in patients with RA excluded patients with diabetes [24, 43].

Although chronic inflammation has long been implicated as a mediator of insulin resistance and β cell failure in patients with diabetes, the literature provides no clear guidance on the use of bDMARDs in patients with RA comorbid with diabetes. Studies of acute cytokine infusion on glucose metabolism in healthy volunteers may be inappropriate models for chronic inflammatory disease, given that they have produced contradictory results: acute infusion of human recombinant IL-6 has resulted in both an increase in fasting glucose concentrations [44] and a decrease in postprandial glucose concentrations with increased insulin sensitivity [45].

To date, the impact of TNFi on HbA1c, insulin sensitivity/resistance or pancreatic β cell function is unclear [46–52]. An infusion of human recombinant TNFα has been shown to increase [53], as well as decrease [54], insulin sensitivity in healthy volunteers. Symptomatic hypoglycaemia has been reported in patients with diabetes and RA or psoriasis who were treated with etanercept [55–58]. Properly designed clinical trials testing the effect of biologic anti-inflammatory drugs inhibiting TNF (e.g. CDP-571, etanercept) are lacking.

The evidence for IL-1 signalling involvement in glucose regulation is more supportive. A recent meta-analysis of > 2900 patients T2D treated with biologics that block IL-1 signalling (anakinra, canakinumab, gevokizumab, LY2189102) demonstrated a significant overall reduction in HbA1c of 0.32%; this included a study of patients with RA and T2D that showed a reduction of > 0.8% [21].

However, no bDMARD has yet been recommended or approved for the treatment of diabetes, although a clinical trial to assess the potential efficacy of anti-IL-6 therapy (tocilizumab) in patients with type 1 diabetes is in progress [59]. A better understanding of the potential differences in the effect of IL-6R blockade vs. IL-1β/TNFα antagonism on common comorbidities in RA should lead to more informed and individually tailored choices in RA disease management.

## Conclusions

In a post hoc analysis of three studies, IL-6R inhibition with sarilumab was associated with a reduction in HbA1c in patients with RA with and without comorbid diabetes that were greater than with placebo or adalimumab and could not be attributed solely to changes in CRP, disease activity or haemoglobin. The safety and efficacy of sarilumab in patients with diabetes were consistent with the results for prespecified patient populations in the individual studies. Prospective randomised clinical trials are needed to evaluate the effects of IL-6R inhibition on glycaemic indices, insulin sensitivity and pancreatic β cell function in patients with comorbid RA and diabetes and determine the clinical relevance of differences in IL-6R, IL-1β and TNF inhibition.

## Supplementary information

**Additional file 1.**

## Data Availability

Qualified researchers may request access to patient level data and related study documents, including the clinical study report, study protocol with any amendments, blank case report form, statistical analysis plan and dataset specifications. Patient-level data will be anonymized and study documents will be redacted to protect the privacy of trial participants. Further details on Sanofi’s data-sharing criteria, eligible studies and process for requesting access can be found at: https://www.clinicalstudydatarequest.com.

## Abbreviations

AE: Adverse event
ALT: Alanine aminotransferase
ACR20: American College of Rheumatology 20%
ANC: Absolute neutrophil count
CCP: Cyclic citrullinated peptide
CI: Confidence interval
CDAI: Clinical Disease Activity Index
csDMARDs: Conventional synthetic disease-modifying antirheumatic drugs
CRP: C-reactive protein
DAS28-CRP: Disease Activity Score(28 joints) using C-reactive protein
HbA1c: Glycosylated haemoglobin
HAQ-DI: Health Assessment Questionnaire Disability Index
IL-6R: Interleukin-6 receptor
IR: Inadequate response
IL: Interleukin
INT: Intolerant
LDL-C: Low-density lipoprotein cholesterol
LS: Least squares
MTX: Methotrexate
MMRM: Mixed-effect model repeat measurement
PBO: Placebo
q2w: Every 2 weeks
RA: Rheumatoid arthritis
SD: Standard deviation
SE: Standard error
SAE: Serious TEAE
*r*_S_: Spearman’s rank correlation coefficient
T2D: type 2 diabetes
TEAE: Treatment-emergent adverse event
TNFi: Tumour necrosis factor inhibitor
TNFα: Tumour necrosis factor-α
ULN: Upper limit of normal
