# Supplementary material for: Interleukin-6 receptor blockade or TNFα inhibition for reducing glycaemia in patients with RA and diabetes: post hoc analyses of three randomised, controlled trials
Source: Arthritis Res Ther. 2020 Sep 9;22:206. doi: 10.1186/s13075-020-02229-5 (PMC7488252; doi:10.1186/s13075-020-02229-5)
Supplement: Supplementary file 1 — Additional file 1. [file 13075_2020_2229_MOESM1_ESM.docx]

**Genovese MC et al. Interleukin-6 receptor blockade or TNFα inhibition for reducing glycaemia in patients with RA and diabetes: post hoc analyses of three randomized, controlled trials**

## Supplementary Tables and Figures

**Figure S1.** Efficacy outcomes at Week 24 in patients with RA with or without diabetes who received sarilumab 150 mg or 200 mg q2w + MTX/csDMARDs in two placebo-controlled trials (A, C, E, G; pooled data) or sarilumab 200 mg q2w monotherapy (B, D, F, H): for ACR20 (A and B), change from baseline in HAQ-DI (C and D), change from baseline in DAS28-CRP (E and F), change from baseline in CDAI (G and H).

**Table S1.** Patient disposition by diabetes status across three phase III studies of patients with RA

**Table S2.** Change from baseline at Week 24 in HbA1c by DAS28-CRP category and diabetes status

**Table S3.** Lack of interaction between change from baseline in HbA1c and (A) baseline oral glucocorticoid use or (B) increase from baseline in haemoglobin at Week 24

**Table S4.** Interaction between change from baseline in HbA1c and baseline HbA1c <7.0% vs ≥ 7.0%, baseline status of diabetes vs no diabetes, baseline oral glucocorticosteroid use, and with or without increase in haemoglobin at Week 24

**Table S5.** Change from baseline in body weight at Week 24 by diabetic status

### **Figure S1.** Efficacy outcomes at Week 24 in patients with RA with or without diabetes who received sarilumab 150 mg or 200 mg q2w + MTX/csDMARDs in two placebo-controlled trials (A, C, E, G; pooled data) or sarilumab 200 mg q2w monotherapy (B, D, F, H): for ACR20 (A and B), change from baseline in HAQ-DI (C and D), change from baseline in DAS28-CRP (E and F) and change from baseline in CDAI (G and H).


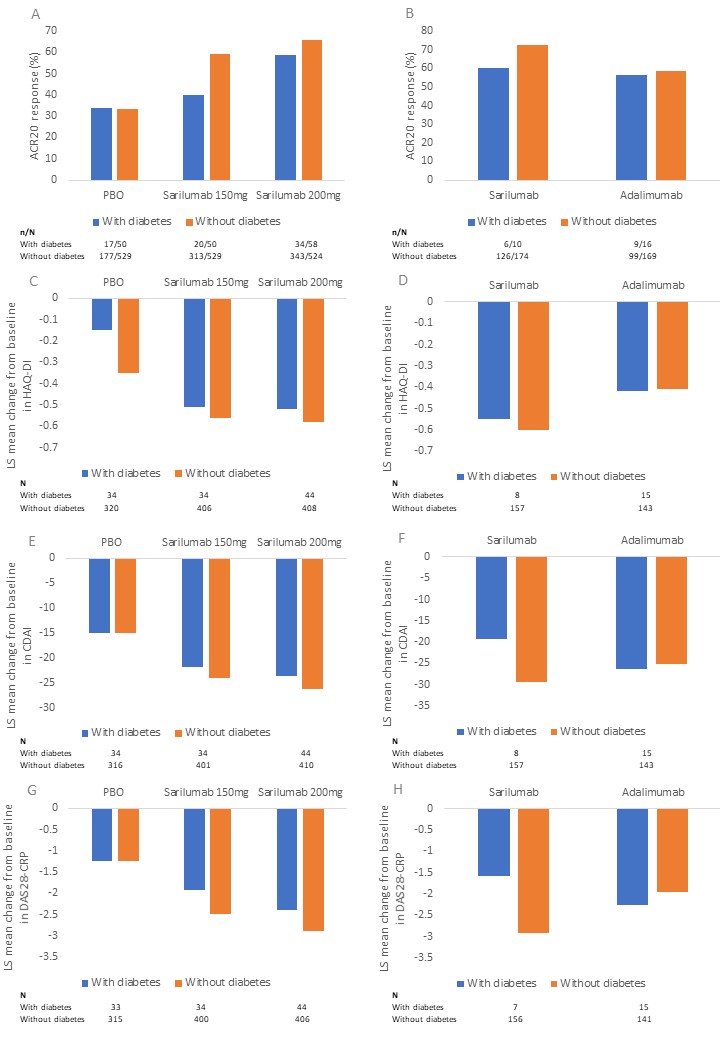


ACR20 = American College of Rheumatology 20% improvement response; CDAI = Clinical Disease Activity Index; csDMARD = conventional synthetic disease-modifying antirheumatic drug; DAS28-CRP = Disease Activity Score 28-C-reactive protein; HAQ-DI = Health Assessment Questionnaire-Disability Index; MTX = methotrexate; q2w = every 2 weeks.

### **Table S1.** Patient disposition by diabetes status across three phase III studies of patients with RA

|  | **Total** | |
| --- | --- | --- |
| **n (%)** | **With diabetes (N** = **184)** | **Without diabetes (N** = **1928)** |
| Randomized and not treated | 0 | 4 (0.2) |
| Randomized and treated | 184 (100) | 1924 (99.8) |
| Completed the study treatment period up to Week 24 | 145 (78.8) | 1618 (83.9) |
| Discontinued during double-blind period | 27 (14.7) | 200 (10.4) |
| Subject’s request for treatment discontinuation | 12 (6.5) | 95 (4.9) |
| Reason for treatment discontinuation |  |  |
| AE | 16 (8.7) | 123 (6.4) |
| Lack of efficacy | 3 (1.6) | 24 (1.2) |
| Poor compliance to protocol | 3 (1.6) | 8 (0.4) |
| Other reasons | 5 (2.7) | 45 (2.3) |
|  | | |

AE = adverse event.

### **Table S2.** Change from baseline at Week 24 in HbA1c by DAS28-CRP category and diabetes status

|  | **Combination therapy with csDMARDs  in TNF-INT/IR patients** | | | **Monotherapy in MTX-INT/IR patients** | |
| --- | --- | --- | --- | --- | --- |
| **Change in HbA1c at Week 24** | **Placebo + csDMARDs** | **Sarilumab 150 mg q2w + csDMARDs** | **Sarilumab 200 mg q2w + csDMARDs** | **Adalimumab 40 mg q2w monotherapy** | **Sarilumab 200 mg q2w monotherapy** |
| **DAS28-CRP < 2.6** | | | | | |
| Patients with a medical history of diabetes or baseline use of antidiabetic medication | | | | | |
| Number | 1 | 3 | 2 | 2 | 0 |
| Mean (SD) | −0.20 (-) | 0.17 (0.50) | −0.55 (0.35) | −0.50 (0.14) |  |
| Median (Q1, Q3) | −0.20 (−0.20, −0.20) | 0.10 (−0.30, 0.70) | −0.55 (−0.80, −0.30) | −0.50 (−0.60, −0.40) |  |
| Patients without diabetes | | | | | |
| Number | 12 | 42 | 49 | 23 | 60 |
| Mean (SD) | 0.38 (0.72) | −0.17 (0.32) | −0.21 (0.29) | −0.05 (0.30) | −0.08 (0.55) |
| Median (Q1, Q3) | 0.15 (0.05, 0.30) | −0.15 (−0.30, −0.10) | −0.20 (−0.40, 0.00) | 0.00 (−0.20, 0.20) | −0.10 (−0.30, 0.00) |
| **DAS28-CRP ≥ 2.6** |  |  |  |  |  |
| Patients with a medical history of diabetes or baseline use of antidiabetic medication | | | | | |
| Number | 14 | 11 | 13 | 12 | 6 |
| Mean (SD) | 0.25 (1.27) | −0.25 (0.56) | −0.55 (0.99) | 0.17 (0.70) | −0.28 (0.57) |
| Median (Q1, Q3) | 0.15 (−0.40, 0.50) | −0.30 (−0.60, 0.40) | −0.30 (−0.70, −0.20) | 0.25 (−0.40, 0.30) | −0.20 (−0.80, 0.30) |
| Patients without diabetes |  |  |  |  |  |
| Number | 70 | 70 | 70 | 111 | 92 |
| Mean (SD) | 0.04 (0.35) | −0.13 (0.32) | −0.32 (0.30) | −0.03 (0.33) | −0.20 (0.32) |
| Median (Q1, Q3) | 0.00 (−0.20, 0.30) | −0.10 (−0.30, 0.10) | −0.30 (−0.50, −0.10) | 0.00 (−0.20, 0.20) | −0.20 (−0.40, 0.00) |

| **DAS28-CRP < 3.2** |  |  |  |  |  |
| --- | --- | --- | --- | --- | --- |
| Patients with a medical history of diabetes or baseline use of antidiabetic medication | | | | | |
| Number | 2 | 5 | 5 | 2 | 0 |
| Mean (SD) | −0.95 (1.06) | −0.16 (0.58) | −0.62 (0.44) | −0.50 (0.14) |  |
| Median (Q1, Q3) | −0.95 (−1.70, −0.20) | −0.30 (−0.50, 0.10) | −0.50 (−0.80, −0.30) | −0.50 (−0.60, −0.40) |  |
| Patients without diabetes |  |  |  |  |  |
| Number | 23 | 54 | 67 | 42 | 92 |
| Mean (SD) | 0.23 (0.61) | −0.14 (0.32) | −0.22 (0.27) | −0.06 (0.31) | −0.14 (0.50) |
| Median (Q1, Q3) | 0.10 (−0.10, 0.30) | −0.10 (−0.30, 0.00) | −0.20 (−0.40, −0.10) | 0.05 (−0.30, 0.20) | −0.20 (−0.40, 0.00) |
| **DAS28-CRP ≥ 3.2** |  |  |  |  |  |
| Patients with a medical history of diabetes or baseline use of antidiabetic medication | | | | | |
| Number | 13 | 9 | 10 | 12 | 6 |
| Mean (SD) | 0.40 (1.19) | −0.16 (0.58) | −0.51 (1.10) | 0.17 (0.70) | −0.28 (0.57) |
| Median (Q1, Q3) | 0.20 (−0.10, 0.50) | −0.30 (−0.50, 0.40) | −0.30 (−0.70, −0.20) | 0.25 (−0.40, 0.30) | −0.20 (−0.80, 0.30) |
| Patients without diabetes |  |  |  |  |  |
| Number | 59 | 58 | 52 | 92 | 60 |
| Mean (SD) | 0.04 (0.33) | −0.15 (0.32) | −0.35 (0.32) | −0.03 (0.33) | −0.16 (0.30) |
| Median (Q1, Q3) | 0.00 (−0.20, 0.30) | −0.10 (−0.30, 0.00) | −0.30 (−0.50, −0.10) | 0.03 (−0.20, 0.20) | −0.20 (−0.35, 0.00) |
| All assessments are set to missing from the time a patient prematurely discontinues study medication. Patients with nonmissing HbA1c and DAS28-CRP values are considered. Number = Number of patients with assessment at both baseline and Week 24. | | | | | |
| csDMARDs = conventional synthetic disease-modifying antirheumatic drug; DAS28-CRP = Disease Activity Score (28 joints) using C-reactive protein; IN = intolerant; IR = inadequate response; MTX = methotrexate; SD = standard deviation; TNF = tumor necrosis factor; Q1 = first quartile; q2w = every 2 weeks; Q3 = third quartile. | | | | | |

### **Table S3.** Lack of interaction between change from baseline in HbA1c and either (A) baseline oral glucocorticoid use or (B) increase from baseline in haemoglobin at Week 24

(A)

| **Study** |  |  |  |  |  |  |
| --- | --- | --- | --- | --- | --- | --- |
| **Combination therapy in TNF-INT/IR patients** | |  |  |  |  | ***p* value for interaction^a^** |
|  | **Subgroup** |  | **Placebo + csDMARD** | **Sarilumab  150 mg q2w + csDMARD** | **Sarilumab  200 mg q2w + csDMARD** | 0.8767 |
|  | With baseline oral  glucocorticoid use | Number | 65 | 91 | 86 |  |
|  |  | Baseline mean (SD) | 5.82 (0.77) | 5.79 (0.72) | 5.73 (0.73) |  |
|  |  | LS mean change (SE)^a^ | 0.09 (0.05) | −0.18 (0.05) | −0.29 (0.05) |  |
|  |  | LS mean diff,  95% CI^a^ |  | −0.27  (−0.41,−0.13) | −0.38  (−0.52,−0.24) |  |
|  |  | *p* value vs. placebo^a^ |  | 0.0001 | < 0.0001 |  |
|  |  |  |  |  |  |  |
|  | Without baseline oral glucocorticoid use | Number | 39 | 41 | 51 |  |
|  |  | Baseline mean (SD) | 5.92 (0.95) | 5.80 (0.71) | 5.75 (0.80) |  |
|  |  | LS mean change (SE)^a^ | 0.08 (0.07) | −0.13 (0.07) | −0.28 (0.06) |  |
|  |  | LS mean diff,  95% CI^a^ |  | −0.21  (−0.40,−0.03) | −0.37  (−0.54,−0.19) |  |
|  |  | *p* value vs. placebo^a^ |  | 0.0245 | < 0.0001 |  |
| **Monotherapy in MTX-INT/IR patients** | |  |  |  |  | ***p* value for interaction^a^** |
|  | **Subgroup** |  | **Adalimumab 40 mg q2w monotherapy** |  | **Sarilumab  200 mg q2w monotherapy** | 0.7438 |
|  | With baseline oral  glucocorticoid use | Number | 86 |  | 80 |  |
|  |  | Baseline mean (SD) | 5.63 (0.62) |  | 5.56 (0.56) |  |
|  |  | LS mean change (SE)^a^ | −0.01 (0.04) |  | −0.16 (0.05) |  |
|  |  | LS mean diff,  95% CI^a^ |  |  | −0.15  (−0.27,−0.03) |  |
|  |  | *p* value vs. placebo^a^ |  |  | 0.0181 |  |
|  |  |  |  |  |  |  |
|  | Without baseline oral glucocorticoid use | Number | 66 |  | 80 |  |
|  |  | Baseline mean (SD) | 5.63 (0.59) |  | 5.54 (0.53) |  |
|  |  | LS mean change (SE)^a^ | −0.01 (0.05) |  | −0.13 (0.05) |  |
|  |  | LS mean diff,  95% CI^a^ |  |  | −0.12  (−0.25,0.01) |  |
|  |  | *p* value vs. placebo^a^ |  |  | 0.0816 |  |
| Note: Number = Number of patients with assessment at both baseline and post baseline visit. ^a^MMRM assuming an unstructured covariance structure. Model = treatment, region, visit, subgroup, treatment-by-visit interaction, treatment-by-subgroup interaction and treatment-by-visit-by-subgroup interaction. Missing data were not imputed.  CI = confidence interval; csDMARD = conventional synthetic disease-modifying antirheumatic drug; HbA1c = glycosylated haemoglobin; INT = intolerant; IR = inadequate response; LS = least squares; MMRM = mixed-effect model repeat measurement; MTX = methotrexate; q2w = every 2 weeks; TNF = tumour necrosis factor-α inhibitor. | | | | | | |

(B)

| **Study** |  |  |  |  |  |  |
| --- | --- | --- | --- | --- | --- | --- |
| **Combination therapy in TNF-INT/IR patients** | | | | | | ***p* value for interaction^a^** |
|  | **Subgroup** |  | **Placebo + csDMARD** | **Sarilumab  150 mg q2w + csDMARD** | **Sarilumab  200 mg q2w + csDMARD** | 0.9362 |
|  | Without an increase in haemoglobin | Number | 57 | 24 | 29 |  |
|  |  | Baseline mean (SD) | 5.93 (0.96) | 5.64 (0.58) | 5.52 (0.86) |  |
|  |  | LS mean change (SE)^a^ | 0.10 (0.06) | −0.17 (0.09) | −0.31 (0.09) |  |
|  |  | LS mean diff,  95% CI^a^ |  | −0.27 (−0.49, −0.05) | −0.41 (−0.61, −0.20) |  |
|  |  | *p* value vs. placebo^a^ |  | 0.0177 | 0.0001 |  |
|  |  |  |  |  |  |  |
|  | With an increase in haemoglobin | Number | 38 | 100 | 102 |  |
|  |  | Baseline mean (SD) | 6.09 (1.14) | 5.63 (0.65) | 5.34 (0.50) |  |
|  |  | LS mean change (SE)^a^ | 0.13 (0.08) | −0.15 (0.05) | −0.32 (0.05) |  |
|  |  | LS mean diff,  95% CI^a^ |  | −0.28 (−0.45, −0.11) | −0.45 (−0.63, −0.28) |  |
|  |  | *p* value vs. placebo^a^ |  | 0.0015 | < 0.0001 |  |
| **Monotherapy in MTX-INT/IR patients** | | | | | | ***p* value for interaction^a^** |
|  | **Subgroup** |  | **Adalimumab 40 mg q2w monotherapy** |  | **Sarilumab 200 mg q2w monotherapy** | 0.6580 |
|  | Without an increase in haemoglobin | Number | 77 |  | 42 |  |
|  |  | Baseline mean (SD) | 5.60 (0.54) |  | 5.37 (0.57) |  |
|  |  | LS mean change (SE)^a^ | −0.02 (0.05) |  | −0.10 (0.06) |  |
|  |  | LS mean diff, 95% CI^a^ |  |  | −0.09 (−0.24, 0.07) |  |
|  |  | *p* value vs. placebo^a^ |  |  | 0.2677 |  |
|  |  |  |  |  |  |  |
|  | With an increase in haemoglobin | Number | 70 |  | 113 |  |
|  |  | Baseline mean (SD) | 5.58 (0.68) |  | 5.41 (0.56) |  |
|  |  | LS mean change (SE)^a^ | −0.04 (0.05) |  | −0.17 (−0.04), |  |
|  |  | LS mean diff,  95% CI^a^ |  |  | −0.13 (−0.25, -0.01) |  |
|  |  | *p* value vs. placebo^a^ |  |  | 0.0351 |  |
| Note: Number = Number of patients with assessment at both baseline and post baseline visit. ^a^MMRM assuming an unstructured covariance structure. Model = treatment, region, visit, subgroup, treatment-by-visit interaction, treatment-by-subgroup interaction, and treatment-by-visit-by-subgroup interaction. Missing data were not imputed.  CI = confidence interval; csDMARD = conventional synthetic disease-modifying antirheumatic drug; INT = intolerant; IR = inadequate response; LS = least squares; MMRM = mixed-effect model repeat measurement; MTX = methotrexate; q2w = every 2 weeks; SD = standard deviation; SE = standard error; TNF = tumour necrosis factor-inhibitor. | | | | | | |

### **Table S4.** Interaction between change from baseline in HbA1c and baseline HbA1c <7.0% vs ≥ 7.0%, baseline status of diabetes vs no diabetes, baseline oral glucocorticosteroid use, and with or without increase in haemoglobin at Week 24

|  | *p* value for interaction^a^ | | |
| --- | --- | --- | --- |
| Change from baseline in HbA1c at Week 24 by subgroups | Model 1^b^ | Model 2^c^ | Model 3^d^ |
| Baseline HbA1c <7.0% vs ≥ 7.0% |  |  |  |
| Monotherapy in MTX-INT/IR patients | 0.0045 | 0.0077 | 0.0022 |
| Combination therapy with csDMARDs in TNF-INT/IR patients | 0.1101 | 0.0779 | 0.0920 |
| Baseline status of diabetes vs no diabetes |  |  |  |
| Monotherapy in MTX-INT/IR patients | 0.0374 | 0.0493 | 0.0277 |
| Combination therapy with csDMARDs in TNF-INT/IR patients | 0.0805 | 0.0746 | 0.0637 |
| With or without baseline oral glucocorticosteroid use |  |  |  |
| Monotherapy in MTX-INT/IR patients | 0.7318 | 0.5382 | 0.8185 |
| Combination therapy with csDMARDs in TNF-INT/IR patients | 0.9794 | 0.9576 | 0.9880 |
| With or without increase in haemoglobin |  |  |  |
| Monotherapy in MTX-INT/IR patients | 0.6095 | 0.6209 | 0.6854 |
| Combination therapy with csDMARDs in TNF-INT/IR patients | 0.9015 | 0.8257 | 0.8661 |

^a^*p* comparing LS mean change from baseline in HbA1c between subgroups using MMRM assuming an unstructured covariance structure. ^b^Model = subgroup, treatment, region, CRP change at week 24, CDAI change at week 24, visit, treatment-by-visit interaction, treatment-by-subgroup, interaction, and treatment-by-visit-by-subgroup interaction. ^c^Model = subgroup, treatment, region, DAS28-CRP change at week 12, visit, treatment-by-visit interaction, treatment-by-subgroup, interaction, and treatment-by-visit-by-subgroup interaction. ^d^Model = subgroup, treatment, region, TJC28 change at week 24, SJC28 change at week 24, PtGA change at week 24, SGA change at week 24, CRP change at week 24, visit, treatment-by-visit interaction, treatment-by-subgroup, interaction, and treatment-by-visit-by-subgroup interaction.

Missing data were not imputed.

CDAI = Clinical Disease Activity Index; CRP = C-reactive protein; csDMARD = conventional synthetic disease-modifying antirheumatic drug; HbA1c = glycosylated haemoglobin; INT = intolerant; IR = inadequate response; MTX = methotrexate; MMRM = mixed-effect model repeat measurement; PtGA = patient global assessment; SGA = subjective global assessment; SJC28 = swollen 28-joint count; TJC28 = tender 28-joint count; TNF = tumour necrosis factor-α inhibitor.

### **Table S5.** Change from baseline in body weight at Week 24 by diabetic status.

|  | **Combination therapy with MTX  in MTX-IR patients** | | | **Combination therapy with csDMARDs  in TNF-INT/IR patients** | | | **Monotherapy in MTX-INT/IR patients** | |
| --- | --- | --- | --- | --- | --- | --- | --- | --- |
| **Body weight at Week 24, n (%)** | **Placebo** | **Sarilumab 150 mg q2w** | **Sarilumab 200 mg q2w** | **Placebo** | **Sarilumab 150 mg q2w** | **Sarilumab 200 mg q2w** | **Adalimumab 40 mg q2w** | **Sarilumab 200 mg q2w** |
| **With diabetes** |  |  |  |  |  |  |  |  |
| Number | 18 | 20 | 29 | 15 | 14 | 15 | 14 | 7 |
| > +5% | 1 (5.6) | 2 (10.0) | 5 (17.2) | 2 (13.3) | 2 (14.3) | 2 (13.3) | 2 (14.3) | 1 (14.3) |
| -5% − ≤+5% | 15 (83.3) | 18 (90.0) | 23 (79.3) | 11 (73.3) | 11 (78.6) | 12 (80.0) | 10 (71.4) | 6 (85.7) |
| ≤-5% | 2 (11.1) | 0 | 1 (3.4) | 2 (13.3) | 1 (7.1) | 1 (6.7) | 2 (14.3) | 0 |
| **Without diabetes** |  |  |  |  |  |  |  |  |
| Number | 234 | 295 | 288 | 84 | 112 | 120 | 146 | 159 |
| > +5% | 25 (10.7) | 49 (16.6) | 53 (18.4) | 6 (7.1) | 11 (9.8%) | 22 (18.3) | 12 (8.2) | 19(11.9) |
| -5% − ≤+5% | 192 (82.1) | 232 (78.6) | 222 (77.1) | 74 (88.1) | 96 (85.7) | 91 (75.8) | 126 (86.3) | 134 (84.3) |
| ≤ -5% | 17 (7.3) | 14 (4.7) | 13 (4.5) | 4 (4.8) | 5 (4.5) | 7 (5.8) | 8 (5.5) | 6 (3.8) |

csDMARD = conventional synthetic disease-modifying antirheumatic drug; INT = intolerant; IR = inadequate response; MTX = methotrexate; q2w = every 2 weeks; TNF = tumour necrosis factor-α inhibitor.
